# Frailty is an independent risk factor of one-year mortality after elective orthopedic surgery: a prospective cohort study

**DOI:** 10.18632/aging.202576

**Published:** 2021-02-26

**Authors:** Xiaoyun Sun, Yuying Shen, Muhuo Ji, Shanwu Feng, Yuzhu Gao, Jianjun Yang, Jinchun Shen

**Affiliations:** 1 Department of Anesthesiology, Women’s Hospital of Nanjing Medical University, Nanjing Maternity and Child Health Care Hospital, Nanjing 210004, China; 2 Department of General Practice, Nanjing First Hospital, Nanjing Medical University, Nanjing 210006, China; 3 Department of Anesthesiology, The Second Affiliated Hospital, Nanjing Medical University, Nanjing 210003, China; 4 Department of Anesthesiology, Jinling Hospital, Medical School of Nanjing University, Nanjing 210002, China; 5 Department of Anesthesiology, Pain and Perioperative Medicine, The First Affiliated Hospital of Zhengzhou University, Zhengzhou 450052, China

**Keywords:** elderly, frailty, orthopedic surgery, mortality

## Abstract

Frailty is associated with perioperative adverse outcomes, especially for the elderly. This study aimed to assess whether frailty was an independent risk factor of one-year mortality in frail patients after elective orthopedic surgery. In this prospective study, three hundred and thirteen patients aged ≥ 65 years, undergoing elective orthopedic surgery were finally included. Frailty assessed by the Clinical Frailty Score (CFS) before the surgery was present in 29.7% (93/313). Among them, 7.7% of patients (24/313) died at one year after surgery. In multivariate logistic analysis, higher CFS (OR = 2.271, 95% CI= 1.472–3.504) was found to be an independent risk factor of one-year mortality after surgery in elderly orthopedic patients. The area under the receiver operating characteristic curve of the model was 0.897 (95% CI 0.834–0.959). In addition, we found higher Charlson comorbidity index (OR= 1.498, 95% CI = 1.082–2.073) was also a significant risk factor. In conclusion, frailty is associated with increased one-year mortality in elderly patients after elective orthopedic surgery, which should be considered as a routine assessment tool in preoperative practice.

## INTRODUCTION

It is predicted that by 2050, about 16% of the global population will be over 65, so the future demand for health care is expected to increase tremendously [1]. Elderly patients make up an ever-increasing proportion of the surgical population, which are associated with increased all-cause morbidity and mortality. However, there is no reliable tool to predict mortality of elderly patients undergoing elective orthopedic surgery.

Frailty is defined as an objective measurement of increased vulnerability and decreased physiological reserve, resulting from the age-associated accumulation of physiological deficits in multiple systems [2]. Frailty in the elderly is accompanied by sarcopenia, loss of function, and disability [3], which is associated with diminished resilience to stressors. Accumulating evidence has suggested that frailty is a risk factor for adverse outcomes, including prolonged hospitalization, increased morbidity and mortality [1, 2, 4, 5]. The Canadian Study of Health and Aging-Clinical Frailty Scale (CSHA-CFS) is developed as a new tool to predict death or institutional care needs in 2005 [6, 7]. This scale is applied to elderly patients participating in the second stage of the CSHA, and has been recognized as a reliable tool to detect frailty in the medical setting [6]. The CFS is simple to perform and has a good correlation with the more thorough frailty index, which has been shown to predict morbidity and mortality in some surgical populations [7]. Meanwhile, the American College of Surgeons (ACS) and the American Geriatrics Society (AGS) jointly recommend frailty assessment as part of a preoperative assessment of the elderly patients. Although the CFS is a simple, less time-consuming and reliable tool, few studies have focused on its application in surgical patients. In addition, the results derived from the preoperative use of the CFS in a population consisting only of elderly patients undergoing elective orthopedic surgery remain to be elucidated.

Therefore, the aim of the present study was to assess whether frailty as assessed by the CFS can predict one-year mortality in elderly patients after elective orthopedic surgery.

## RESULTS

### Baseline characteristics

**Figure 1:**
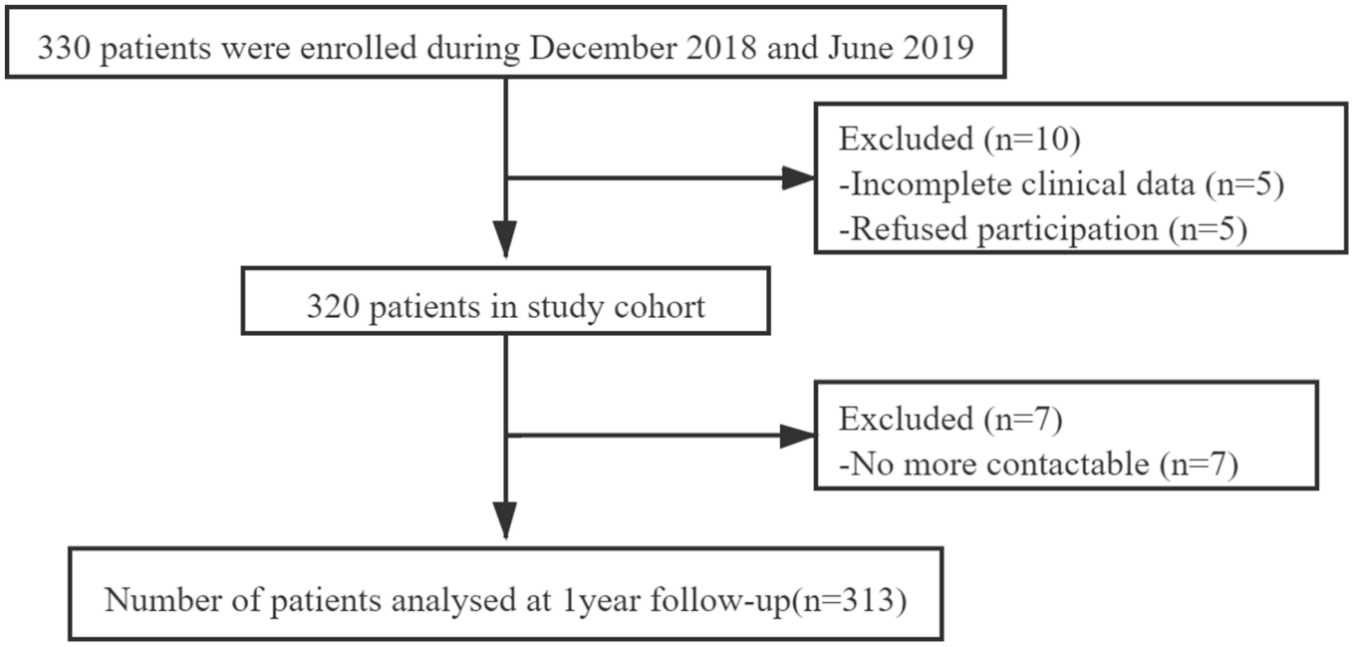
**Patient enrollment flow chart.**

Three hundred and thirteen patients were finally included in our study (Figure 1). Baseline characteristics were presented in Table 1. Patients who died had advanced age (80.7 ± 7.0 years) compared with patients who survived (73.3 ± 6.9 years) (*P* < 0.001). During one-year follow-up, 24 patients (7.7%) died. There was no statistically significant difference between males and females in one-year mortality (*P* = 0.079). Patients who died had a higher CFS points [6 (4, 7)] compared with patients who survived [3 (2, 5)] (*P* < 0.001). Moreover, patients who died had lower body mass index (BMI) and mini-mental state examination (MMSE) compared with patients who survived (all *P* < 0.05). There were significantly higher American Society of Anesthesiologists (ASA) Grade, Charlson comorbidity index, basic activities of daily living (BADL) and instrumental activities of daily living (IADL) in patients who died than patients alive (all *P* < 0.05). Patients who died used more prescription drugs compared with patients who survived (*P* = 0.029).

**Table 1:** Characteristics of elderly patients undergoing elective orthopedic surgery according to survival status within one-year.

**Variables**	**Patients alive** **N=289**	**Patients died** **N=24**	**All patients** **N=313**	**P**
**Preoperative variables**				
**Age, years, mean ±SD**	73.3 ± 6.9	80.7 ± 7.0	73.9 ± 7.2	< 0.001
**Sex (female)**	206 (71.3%)	13 (54.2%)	219 (70.0%)	0.079
**BMI (kg/m2), median (IQR)**	24.5 (22.0, 26.7)	22.5 (20.2, 24.5)	24.3 (21.9, 26.5)	0.009
**Education, years, median (IQR)**	6.0 (0.0, 9.0)	5.0 (0.0, 12.0)	6.0 (0.0, 9.5)	0.966
**Prescription drugs, n**	2 (1, 3)	3 (1, 5)	2 (1, 3)	0.029
**ASA Grade**				< 0.001
ASA I	7 (2.4 %)	0 (0.0 %)	7 (2.2%)	
ASA II	174 (60.2 %)	1 (4.2 %)	175 (55.9%)	
ASA III	105 (36.3 %)	19 (79.2 %)	124 (39.6%)	
ASA IV	3 (1.0 %)	4 (16.7 %)	7 (2.2%)	
**MMSE, points, median (IQR)**	21 (17, 25)	15 (12, 18)	21 (17, 25)	< 0.001
**Charlson comorbidity index, points, median (IQR)**	1 (0, 2)	3 (2, 5)	1 (0, 2)	< 0.001
**BADL, points, median (IQR)**	2 (0, 7)	11 (5, 12)	2 (0, 8)	< 0.001
**IADL, points, median (IQR)**	8 (6, 12)	17 (12, 21)	8 (6, 13)	< 0.001
**CFS, points, median (IQR)**	3 (2, 5)	6 (4, 7)	3 (2, 5)	< 0.001
**Alb (g/L), mean ±SD**	38.1 ± 4.0	36.0 ± 3.5	37.9 ± 4.0	0.014
**Hb (g/L), mean ±SD**	126.1 ± 16.7	112.5 ± 20.7	125.0 ± 17.4	< 0.001
**PLT (×109/L), mean ±SD**	212 ± 64	182 ± 63	210 ± 64	0.030
**CRP (g/L), median (IQR)**	2.2 (0.5, 15.7)	23.3 (1.2, 56.5)	2.3 (0.5, 18.9)	0.004
**IL-6 (ng/L), median (IQR)**	7.3 (2.8, 15.5)	20.5 (10.6, 33.5)	8.1 (2.9, 18.6)	0.001
**Intraoperative variables**				
**Amount of blood loss (ml), median (IQR)**	200 (120, 250)	265 (200, 350)	200 (120, 280)	0.007
**Duration of surgery (min), median (IQR)**	75 (60, 95)	78 (60, 106)	75 (60, 95)	0.660
**Type of anesthesia (general anesthesia)**	72 (24.9%)	5 (20.8%)	77 (24.6%)	0.656
**Postoperative variables**				
**Post-operative delirium**	41 (14.2%)	14 (58.3%)	55 (17.6%)	< 0.001
**Length of stay, days, median (IQR)**	7 (6, 9)	10 (7, 13)	7 (6, 9)	0.001
**30-d Readmission**	19 (6.6 %)	5 (23.8 %)	24 (7.7 %)	0.015
**30-d CCI, points, mean ± SD**	0.0 (0.0, 25.7)	38.2 (23.0, 56.3)	0.0 (0.0, 29.6)	< 0.001

Abbreviation: SD, Standard deviation; IQR, interquartile range; BMI, body mass index; ASA Grade, American Society of Anesthesiologists Grade; MMSE, mini-mental state examination; BADL, basic activities of daily living; IADL, instrumental activities of daily living; CFS, the Clinical Frailty Score; Alb, albumin; Hb, hemoglobin; PLT, platelet; CRP, C-reactive protein; IL-6, interleukin-6; 30-d Readmission, 30-day readmission; 30-d CCI, 30-day comprehensive complication index.

Albumin (Alb), hemoglobin (Hb), and platelet (PLT) were lower in patients who died than patients alive (all *P* < 0.05). However, C-reactive protein (CRP) and interleukin-6 (IL-6) in patients who died were higher compared with patients alive (all *P* < 0.05). Blood loss was significantly higher in patients who died compared with patients alive (*P* = 0.007). In addition, 58.3% of patients who died had post-operative delirium compared with 14.2% patients alive (*P* < 0.001). Median length of stay for patients who died was longer compared with patients alive (*P* = 0.001). Moreover, the rate of 30 d-readmission in patients who died was significantly higher than patients alive (*P* = 0.015). There was a statistically significant association between increased one-year mortality and higher 30 d-comprehensive complication index (30 d-CCI) (*P* < 0.001).

### Predictive factors of one-year mortality

**Figure 2:**
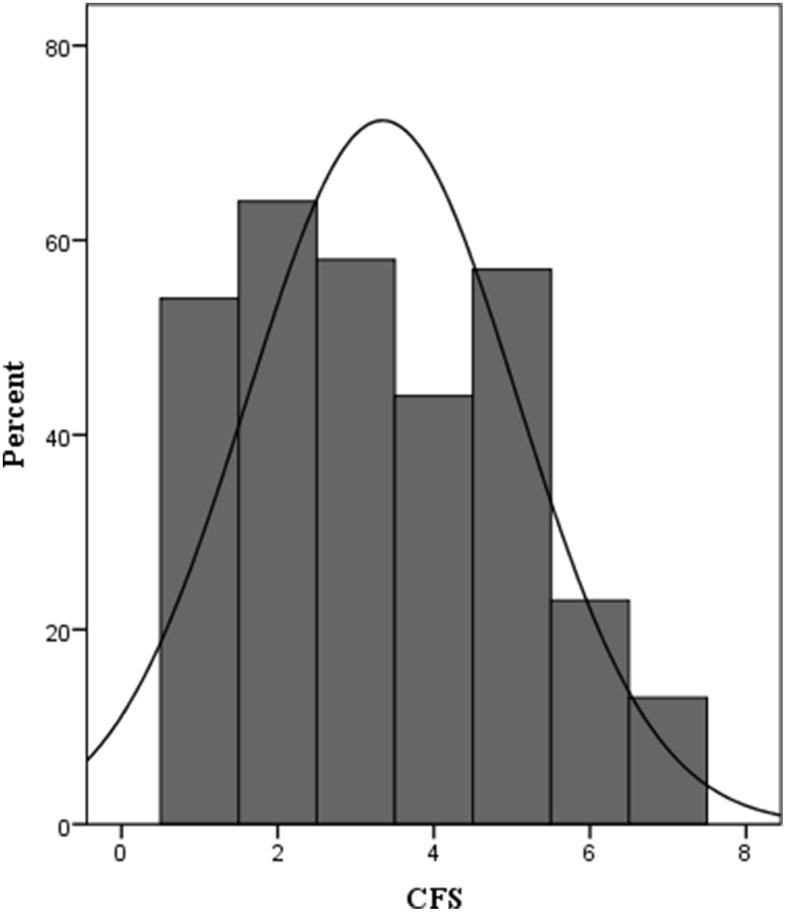
**Distribution of CFS at admission.** CFS, the Clinical Frailty Score, CFS ≥ 5 (frail).

**Figure 3:**
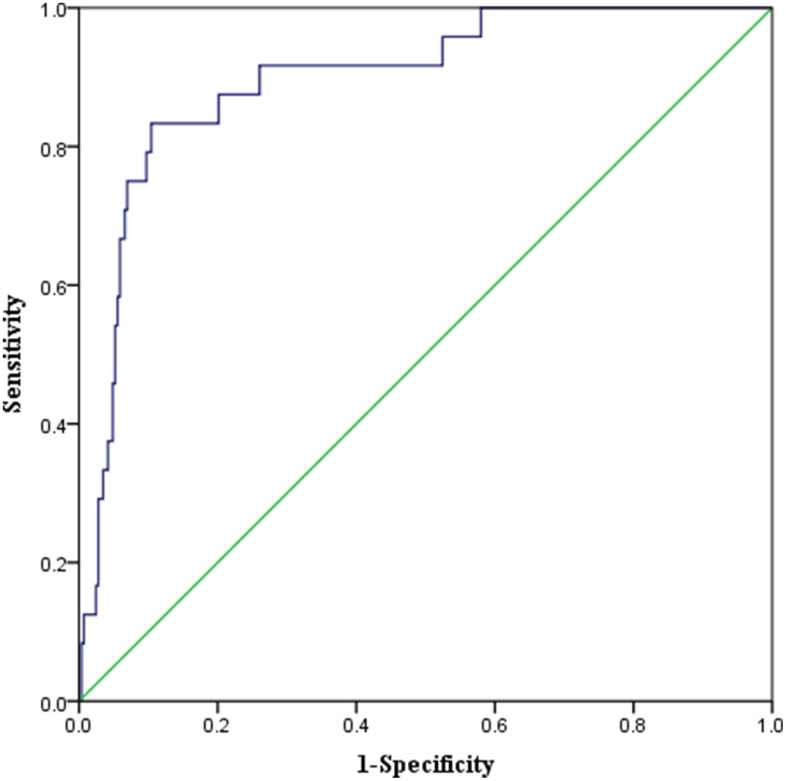
**ROC Curve of the predictive model of one-year mortality in elderly patients undergoing elective orthopedic surgery.** AUC 0.897 (*P* < 0.001, 95% CI 0.834–0.959).

The mean CFS score of all participants was 3 ± 2 points. Ninety-three patients were identified to be frail with a CFS score ≥ 5 (Figure 2). Univariate logistic analysis showed that twelve factors were significantly associated with one-year morality. The multivariate logistic analysis identified higher baseline CFS (OR = 2.271, 95% CI = 1.472–3.504) and Charlson comorbidity index (OR = 1.498, 95% CI = 1.082–2.073) as significant risk factors of one-year mortality after elective orthopedic surgery (Table 2). The receiver operating characteristic (ROC) curve derived from CFS had an area under the curve (AUC) of 0.897 (95% CI: 0.834–0.959) (Figure 3).

**Table 2:** Factors affecting one-year mortality: multivariate analysis.

**Predictive factors**	**OR**	**95% CI**	**P**
CFS	2.271	1.472–3.504	<0.001
Charlson comorbidity index	1.498	1.082–2.073	0.015
Age	1.050	0.941–1.171	0.385
BMI	0.952	0.818–1.107	0.523
Prescription drugs	1.056	0.829–1.346	0.659
MMSE	0.931	0.824–1.051	0.247
IADL	0.905	0.748–1.095	0.303
Alb	1.134	0.985–1.305	0.081
Hb	0.972	0.943–1.002	0.064
PLT	0.993	0.984–1.001	0.084
IL-6	1.011	0.989–1.033	0.336
Amount of blood loss	1.002	0.998–1.006	0.332

*OR* = odds ratio.

Abbreviation: CFS, the Clinical Frailty Score; BMI, body mass index; MMSE, mini-mental state examination; IADL, instrumental activities of daily living; Alb, albumin; Hb, hemoglobin; PLT, platelet; IL-6, interleukin-6.

## DISCUSSION

With the development of surgical, anesthetic and intensive care technologies, more and more elderly patients require surgery. It was estimated that approximately 53% of operations were performed on patients older than 65 years [8]. Of the surgical patients selected in the National Surgical Quality Improvement Program database, 7% of patients underwent orthopedic surgery [9]. In addition, the incidence of frailty is substantially higher in high-risk populations, especially for elderly patients having major orthopedic surgery (41%) [10, 11]. In the present study, we showed that frailty identified by the CFS is significantly associated with increased one-year mortality in elderly patients undergoing elective orthopedic surgery.

Preoperative assessment is vital for hospitalized elderly patients at risk of adverse outcomes. However, there are currently limited screening tools to predict adverse postoperative complications in vulnerable individuals. Evaluation of preoperative risk is usually performed by assessing the ASA Grade. These tools are limited because they cannot cover the complexity of the elderly [12]. Therefore, the existence of frailty has become a method for clinicians to describe the physiological reserve of the elderly.

In recent years, frailty as a screening tool to predict the outcome after major surgery has attracted more and more attention. It can measure the physiological reserve and the ability of the patient to manage surgical stress accurately and easily. Although the ACS recommends a preoperative assessment of frailty, one gold standard to measure the degree of frailty has not yet existed. Frailty is a complex state composed of multiple domains, with incremental increases in frailty scores predicting greater risk of one-year mortality. Although there are many tools for the assessment of frailty, the CFS is a useful tool that is widely used to predict the mortality across studies. For example, the presence of frailty calculated using the progressive CFS in older adults undergoing emergency laparotomy was associated with greater risks of postoperative mortality and was independent of age [13]. In addition, the CFS was a negative prognostic factor for cancer-specific survival in elderly patients with hepatocellular carcinoma who underwent hepatectomy [14]. A recent systematic review and meta-analysis indicated that the CFS was associated with increased mortality, with the largest effect size of any instrument [15]. Moreover, the CFS is reported to improve all measures of predictive performance across outcomes compared to the Fried Phenotype and Frailty Index. Thus, CFS should be recommended as a preferred tool for frailty assessment in preoperative practice [16].

Identifying frail patients, especially when undergoing an elective orthopedic surgery, may be a crucial step in coping with postoperative outcomes and reducing one-year mortality rate. In our previous study, we used Edmonton Frail Scale for frailty assessment, which suggested that frailty is an independent risk predictor of postoperative short-term complications after elective orthopedic surgery, even after adjusting for important confounding factors [17]. In the present study, we used another tool for frailty assessment by the CFS and found frailty is an independent risk factor for one-year mortality after elective orthopedic surgery. Our results are supported by other studies, which suggested that frailty is associated with adverse postoperative outcomes, including prolonged hospital stay, institution discharge, and increased mortality [11, 18–21]. However, the majority of these previous studies have focused on the association between frailty and mortality among community-dwelling older persons or residents in acute care settings [20–21], rather than on those undergoing orthopedic surgery. In addition, these studies also used other methods for frailty assessment, which is based on strength, gait, body composition, and fatigue [21] or a frailty index (FI) using 36 health-related deficits/variables [20]. Indeed, it has been suggested that a more targeted strategy to improve postoperative outcomes may be facilitated using the CFS, a simple, less time-consuming and reliable tool [22].

Some limitations have been noted in our study. Firstly, patients with severe comorbidities might introduce a selection bias and thereby overestimated the prevalence of frailty. Some studies have shown that current frailty assessments may be affected by bias and are less feasible in very frail or ill patients [23, 24]. Since these tools for frailty rely on physical assessment tests, some studies have proposed the measurement of sarcopenia as an objective, quantitative replacement for frailty in various surgical procedures [25, 26]. Nevertheless, we believed that measurement of sarcopenia is a time-consuming and not readily available process, requiring additional hospitalization costs. Another limitation of this study was related to its single-center nature. Therefore, the generalizability of our results is not guaranteed, and the results may be affected by unmeasured confounding variables. Finally, given that one-year mortality was our primary endpoint, future studies should consider other patient-centered outcomes, including long-term postoperative complications and quality of life assessment.

In the present study, we have proved that frailty identified by the CFS is associated with increased one-year mortality after elective orthopedic surgery in elderly patients. Thus, frailty has additional value compared with traditional risk assessment tools and may be used to optimize the risk stratification of this elderly patient population.

## MATERIALS AND METHODS

### Study design and data collection

We finally evaluated 313 consecutive adult patients over 65 undergoing elective orthopedic surgery from December 2018 through July 2020 in Jinling Hospital. The flowchart of data collection was shown in Figure 1. The prospective cohort study was registered online at https://www.clinicaltrials.gov/ (NCT03792373) and approved by the Ethics Committee of the Jinling Hospital. Informed consents of all patients were obtained. Data of frailty based on the CFS were collected using an interview and clinical examination. Clinical and demographic characteristics were collected by reviewing medical records. Follow-up data for 30 days and one year after surgery were obtained through telephone.

### Frailty assessment

Frailty of each patient at admission was evaluated by the CFS developed by the Canadian Study of Health and Ageing (Table 3) [13]. The CFS is a 7-point comprehensive frailty scale based on clinical assessment of physical activity, energy, mobility and function. This 7-point progressive score was developed within an older adult population and is based on the clinical judgment that patients were considered as non-frail (1 to 4 points), or frail (5 to 7 points). The CFS has been found to be a valid and reproducible score that is simple to understand and apply.

**Table 3:** The clinical frailty score (CFS) developed by the Canadian study of health and ageing (CSHA).

1—Very fit	Robust, active, energetic, well motivated, and fit; these people commonly exercise regularly and are in the most fit group for their age.
2—Well	Without active disease, but less fit than people in category 1.
3—Well, with treated comorbid disease	Disease symptoms are well controlled compared with those in category 4.
4—Apparently vulnerable	Although not frankly dependent, these people commonly complain of being ‘‘slowed up’’ or have disease symptoms.
5—Mildly frail	With limited dependence on others for instrumental activities of daily living.
6—Moderately frail	Help is needed with both instrumental and noninstrumental activities of daily living.
7—Severely frail	Dependent on others for activities of daily living, or terminally ill.

### Clinical data selection

Besides the CFS and one-year mortality, routine clinical data were collected pre-, intra- and postoperatively, including preoperative parameters such as age, sex, BMI, educational years, prescription drugs, ASA Grade, MMSE [27], Charlson comorbidity index [28], BADL [29], IADL [30], Alb, Hb, PLT, CRP, IL-6; intraoperative parameters such as blood loss, duration of surgery, type of anesthesia; and postoperative parameters and outcomes including post-operative delirium [31], length of stay, 30 d-readmission and 30-d CCI [32].

### Statistical analysis

Statistical analysis was performed using SPSS version 23.0 software. Continuous variables were presented as mean ± standard deviation (SD) or median and interquartile range (IQR) when categorical variables were presented as number (percentage). Data with skewed distribution was evaluated using Mann-Whitney U test and the χ2 test was used to compare categorical data between groups. We performed a logistic regression model referring to the other's previous cohort study [33], using the Wald Backward method to identify which factor was an independent risk factor of one-year mortality after elective orthopedic surgery. Variables with *P<*0.10 on the basis of the univariate logistic regression analysis were included in multiple logistic regression analyses, and the occurrence of one-year mortality as dependent variable. In addition, a ROC curve analysis was performed to check the predictive value of the model. *P* <0.05 was considered statistically significant. In this study, the number of patients to be enrolled was determined on the basis of the number of predictive parameters inserted into the multivariate analysis. The general empiric rule suggested that the relationship between the total number of events and the number of groups of explicative variables had to be at least ten for each type of variable [33]. On the basis of these considerations, it was estimated to include at least 230 patients.
